# Hybridization of green synthesized silver nanoparticles with poly(ethylene glycol) methacrylate and their biomedical applications

**DOI:** 10.7717/peerj.12540

**Published:** 2022-01-17

**Authors:** Natasha Anwar, Abbas Khan, Mohib Shah, John J. Walsh, Samreen Saleem, Zeeshan Anwar, Sobia Aslam, Muhammad Irshad

**Affiliations:** 1 Chemistry Department, Abdul Wali Khan University Mardan, Mardan, Pakistan; 2 Botany Department, Abdul Wali Khan University Mardan, Mardan, Pakistan; 3 School of Pharmacy and Pharmaceutical Sciences, University of Dublin, Trinity College, Dublin, Ireland; 4 Faculty of Allied Health Sciences & Technology, Women University Swabi, Swabi, Pakistan; 5 Pharmacy Department, Abdul Wali Khan University Mardan, Mardan, Pakistan

**Keywords:** Catalytic reduction, 4-nitrophenol, Ca-AgNPs, PEGMA capped AgNPs, PC-3 human prostate adenocarcinoma cell line

## Abstract

In the present research, a rapid, simple and efficient green method is used for the incorporation of silver nanoparticles (AgNPs) into poly(ethylene glycol) methacrylate (PEGMA) to create biocatalysts with excellent properties for pharmaceutical purpose. In the first phase, *Caralluma tuberculata* capped AgNPs (Ca-AgNPs) were prepared using green synthetic approach and in the second phase *Caralluma tuberculata* capped AgNPs were hybridized with poly(ethylene glycol) methacrylate to form PEGMA-AgNPs. Both the virgin (naked or uncapped) and polymer-capped materials were characterized spectroscopically and their results were compared. Fourier transform infrared spectroscopy showed no new peak after the capping procedure, showing that only physical interactions takes place during capping. After PEGMA capping, the spectra of the AgNPs red shifted (from 450 nm to 520 nm) and the overall particle size of AgNPs increased. Catalytic activity of the nanoparticles and hybrid system were tested by choosing the catalytic reduction of 4-nitrophenol (4-NP) as a model reaction. Both synthesized NPs and polymer capped NPs exhibits catalytic activity for the reduction of 4-NP to 4-aminophenol. The polymer hybrid exhibits remarkable antiproliferative, antioxidant, cytotoxic, antidiabetic and antileishmanial activities.

## Introduction

**Figure 1:**
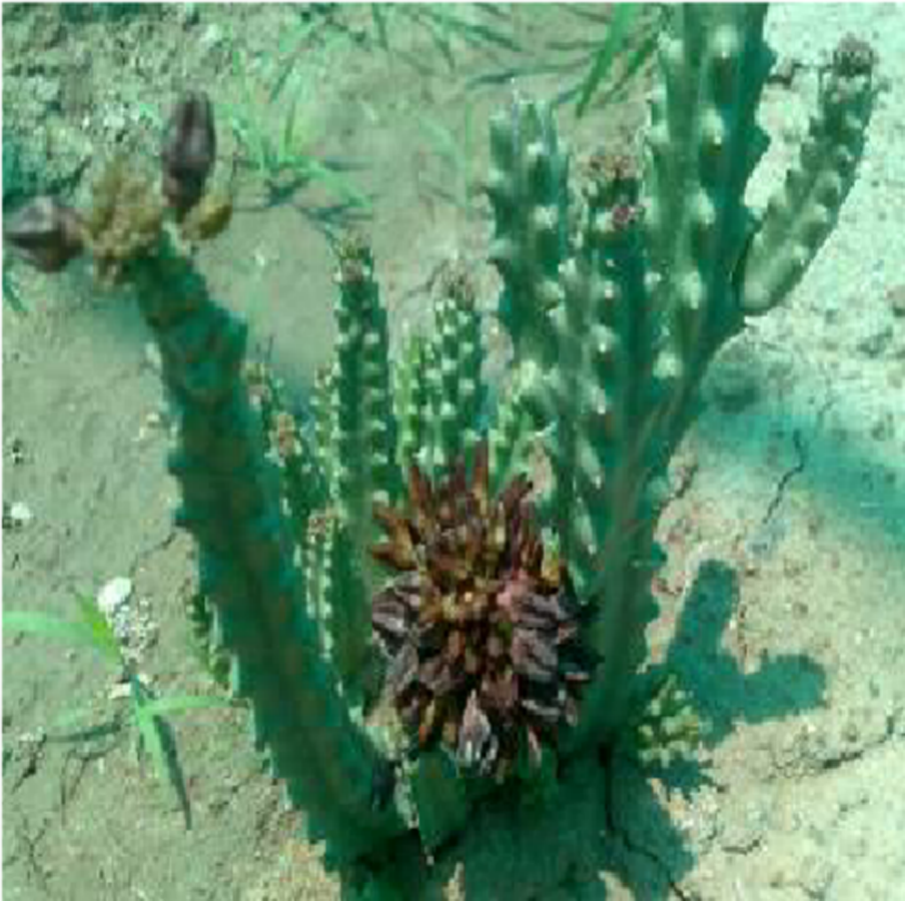
*Caralluma tuberculata*.

Recently, metal nanoparticles have been a subject of significant ongoing studies because of their distinctive properties and potential applications in many areas such as sensors, catalysts, electronics, dye absorption (Mittal, Morajkarppalalili & Alhassan, 2020) and medicine (Xia, Yang & Campbell, 2013; Jang et al., 2010; Anastopoulos et al., 2018). Among the various metal NPs, AgNPs have received much attention due to their attractive shape, size and environment-dependent properties which are totally changed from those of bulk materials (Ramasamy et al., 2012). For the synthesis of AgNPs various chemical, physical and biological methods have been used (Ovais et al., 2016; Iravani et al., 2014) but the NPs prepared by green way are environmental friendly and cost effective. They have low toxicity because all plants have medicinal properties. The other reason is that conventional methods for nanoparticle synthesis usually require harmful reductants such as sodium borohydride or hydrazine and many steps in the synthesis procedure including heat treatments, often producing hazardous by-products. In order to reduce the environmental impact of nanoparticle synthesis, greener routes have been investigated for over a decade (Collins, 2017). Green chemistry should aim at thwarting waste, minimizing energy use, employing renewable materials, and applying methods that minimize risk. The three main concepts for the preparation of nanoparticles in a green synthesis approach are the choice of the solvent medium (preferably water), an environmentally friendly reducing agent, and a nontoxic material for the stabilization of the nanoparticle (Raveendran, Fu & Wallen, 2003). Plants provide the most suitable environment for the preparation of NPs Plants provide the most suitable environment for the preparation of NPs (Ovais et al., 2016). The NPs prepared by chemical method comparatively not suitable for biological activities due its toxicity because hazardous chemical are used (Taheriniya & Behboodi, 2016). So we used a plant based method for the preparation of NPs. For this purpose *Caralluma tuberculata* was used. *Caralluma tuberculata* belongs to family Asclepiadaceae and distributed in dry parts of the world. It has two species *Caralluma tuberculata* and *Caralluma edulis* (Fig. 1). *Caralluma tuberculata* is a leafless, succulent herb and grown in wild. It is a famous traditional medicinal plant in the northern part of Pakistan. It is used as food supplement in Pakistan especially for diabetics (Anwar et al., 2016; Ahmad et al., 2014). Also it is used as treatment for various ailments such as jaundice, stomachic, rheumatism, hepatitis A. While bare AgNPs have shown promise, they are stable and aggregation occurs. Aggregation results in the reduction of various applications such as catalytic and biological activities (Stevanović et al., 2012). To address this issue considerable attention in this area is aimed at trying to control long-term colloidal stability (Zeng et al., 2011), shape (Gawande et al., 2016) and size (Zhu, Li & Xu, 2013) of AgNPs. Polyelectrolytes (Radziuk et al., 2007; Dalavi et al., 2020), dendrimers (Astruc, Ornelas & Ruiz, 2008; Fox, 2020), microgels (Begum et al., 2017; Naseem et al., 2020), block copolymers (Seo et al., 2013; Jia et al., 2020), surfactants (Ding et al., 2014; Rajan, Sharma & Sahu, 2020) and polymer brushes (Chen et al., 2010; Barsbay et al., 2020) are usually used for this purpose. Biodegradable polymers are considered to be an important material because of the applications in various fields such as food (Wei et al., 2020), agriculture (Pawlak-Kruczek et al., 2020), polymeric nanocomposites (Siwal et al., 2020), medical appliances (Ratajczak & Stobiecka, 2020), packaging(Nechita, 2020), building materials (Sun, He & Li, 2020), consumer products (Zhong et al., 2020), industry and efficient carriers for solubilizing and stabilizing various hydrophobic drugs (Rani et al., 2019). In polymeric nanocomposites, highly homogenous materials results due to the combination of polymer and NPs resulting in the growth of novel nanomaterials with excellent and unique properties.

Incorporation of NPs into the polymer matrix not only stabilizes the NPs, but also improve its functional assembly (Yang et al., 2018). Here we present a unique approach to prepare polymer/nanoparticles composites through *ex-situ* reverse micellization technique by dispersing pre-made green AgNPs directly into the polymer to form composites. Then Ca-AgNPs and poly(ethylene) glycol methacrylate capped AgNPs (PEGMA-AgNPs) are used in the catalysis of 4-NP. The polymer composites shows higher catalytic activity as compared to bare AgNPs. Polymer hybrid exhibits remarkable antiproliferative, antioxidant, cytotoxic, antidiabetic and antileishmanial activities.

## Material and Methods

All analytical grade reagents used in this study were purchased from Merck. These included AgNO_3_(1mM), *chloroform ,* ethanol, benzene, n-hexane, distilled water, 4-nitrophenol (4-NP), sodium borohydride and *Triton X - 10* 0. Poly (ethylene glycol) methacrylate (PEGMA, Mn∼526 g/mol), Formula:H_2_C =C(CH_3_)CO(OCH_2_CH_2_)nOH were bought from Sigma-Aldrich (Milwaukee, WI, USA). Fresh *Caralluma tuberculata* was collected in district Mardan, Pakistan. All glassware used in the preparation of all metal salt solutions and hybrid organic and inorganic matrices were cleaned with aqua regia and washed thoroughly with deionized water before use. All UV-visible spectra were measured with a Shimadzu UV-2550 spectrophotometer. Fourier transform infrared (FTIR) spectra were recorded using Nicolet 6700 FT-IR instrument (Thermo Scientific). The solution of nanoparticles and polymer capped were dropped onto copper grids to prepare specimens for transmission electron microscopic (TEM) observation which was performed in a JEM 2100F with a field-emission gun operating at 200 kV. Dynamic light scattering (DLS) study was done using a model BI-200SM instrument (Brookhaven Instrument Corporation) (Anwar et al., 2021).

### Preparation of *Caralluma tuberculata* extract

Fresh *Caralluma tuberculata* was collected from district Mardan, Pakistan. The whole plant was dried and 15 g was taken and 100 ml distilled water was added. It was boiled for 15 min and was filtered. Filtered plant extract was kept at room temperature until required for use.

### Preparation of AgNO_**3**_ Nanoparticles

The AgNO_3_ solution was prepared using distilled water. The plant extract and silver solution were mixed with different salt to plant ratios of 1:1, 2:1, 3:1, 4:1, 5:1, 6:1 and 7:1 respectively. The best optimized ratio, having good SPR in characteristic region of AgNPs, selected for further studies was 5:1. The fine particles so synthesized in solution form were centrifuged at 12000–14000 rpm and the solid samples were collected and then stored in air tight bottles for further characterization and other studies of this project.

### Preparation of PEGMA-coated silver nanoparticles

The surface was functionalized by adding 5 mg of nanoparticles with 1 mL of *Triton X - 100* and 10 mL of mixture of n-hexane and benzene (3:7) in a flask, sonicated for 20 min. The solution was left for an hour on stirring and PEGMA solution (540 µL of PEGMA, Mn 526, dissolved in 100 µL water) was added into it and stirred for 24 h. After this time the volume of the solution was brought up to 45 mL using ethanol and then centrifuged (14000 rpm for 30 min). The supernatant was discarded, the pellet resuspended using water (2 mL) which was followed by a further centrifugation step (14000 rpm, 30 min). The resulting pellet was then characterized.

### Catalytic Activity of nanoparticles and PEGMA capped nanoparticles

The catalytic activity of nanoparticles and polymer hybrid nanoparticles were studied using reduction of 4-NP into 4-AP in aqueous medium. For a representative catalytic experiment a 50 mL solution of 1 mM 4-NP was prepared in a flask and then 0.18 g of NaBH_4_ and 50 mg of catalyst (Ca-AgNPs and PEGMA-AgNPs separately) was added to this solution and the reactions were allowed to occur at room temperature (25 °C). As mentioned the progress of reduction of 4-nitrophenol to 4-aminophenol was regularly monitored by UV-Visible spectrophotometer and for this purpose 0.2 mL was taken in a cuvette from the reaction mixture at different time intervals each of one minute and UV-Visible spectrum was taken. Absorbance was recorded at *λ* = 400 nm until the absorbance did not change at all.

### Cell line

The PC-3 human prostate cancer cell line were obtained from LGC Standards, UK and cultured in F12K medium added with sodium pyruvate of 0.02 mg/mL, penicillin/streptomycin 10 mL/L solution and 10% FBS. Cell cultures were kept in a humidified atmosphere, containing 5 % CO_2_ at 37 °C.

### Cell maintenance and sub-culture

NuAire class II biosafety cabinet was used for cell culture experiments and aseptic techniques were followed to prevent contamination. Cell culture flasks of 75 cm^2^ were used for PC-3 cells culture, using 15 mL of the appropriate complete medium. Olympus CKX41 inverted microscope was used daily to monitor cells (Mason Technology, Ireland).

### Cell proliferation assay

Inhibition of cell growth was evaluated by MTT assay. 5 ×10^4^ cells/well PC-3 cells (180 µL) were added into 96-well plates and incubated at 37 °C in a CO2 incubator for 24 h after which they were treated with Ca-AgNPs and PEGMA capped AgNPs at concentrations in the range of 5 to 100 µg/mL, respectively for 72 h at 37 °C. Distilled water was used as a control. The culture medium was removed and substituted by 200 µL of 0.5 mg/mL MTT solution in complete medium after the incubation period. The solution in each well was replaced by 180 µL of DMSO after 4 h incubation to solubilize the formazan crystals that formed. The absorbance values were read at 584 nm using a FLUOstar OPTIMA microplate reader (BMG Labtech) and calculated the viable cells as by dividing the absorbance of treated cells by the control. Each sample concentration was conducted in duplicate and each experiment was repeated. Graph Pad Prism software was used to plot nonlinear regression graph between cell inhibition (%) and log 10 concentration and IC_50_. Nonlinear regression graph was plotted between cell inhibition (in percentage) and Log10 concentration and IC_50_ values ± standard error of mean (SEM).

% growth inhibition = 100-[(mean Abs of test compound)/(mean Abs of control)]*100.

### DPPH free radical scavenging assay (Antioxidant assay)

The antioxidant activity of Ca-AgNPs and PEGMA capped AgNPs was determined using DPPH (2, 2-Diphenyl-1-Picrylhydrazyl) free radical scavenging assay (Obied et al., 2005). The decrease in the absorption of the DPPH solution after the addition of an antioxidant was measured at 517 nm. Ascorbic acid was used as positive control. A 0.1 mM DPPH solution was prepared by dissolving 4 mg of DPPH in 100 ml of ethanol. Final concentrations of 200, 66.6, 22.2 and 7.4 µg/ml of Ca-AgNPs and PEGMA capped NPs were made up by adding 40 µl of Ca-AgNPs and PEGMA capped NPs in water and making upto 3ml with 2.96 ml DPPH (0.1 mM). The negative control solution consisted of 40 uL of distilled water and 2.96 mL of 0.1 mM DPPH. The reaction mixture was incubated in the dark at room temperature for 30 min. After incubation, the absorbance of the mixture was read at 517 nm (Vidhu & Philip, 2015) The percentage radical scavenging activity of the Ca-AgNPs and PEGMA capped NPs was calculated using the following formula,% Scavenging = [(A-B)/A] ×100

where: A = Absorbance of negative control

B = Absorbance of test sample.

### Brine shrimp cytotoxicity assay

The brine shrimp was used to test cytotoxicity of Ca-AgNPs and PEGMA capped AgNPs (Ul-Haq et al., 2012). Eggs of brine shrimp (*Artemia salina*) (Ocean Star Inc., USA) were hatched within a square box by developing a synthetic seawater environment by blending commercial salt in double distilled water. After 24 h, brine shrimp larvae (Phototropic nauplii) were transferred to glass vial using a Pasteur pipette (Mahmood et al., 2019). 25 µL solutions (200, 66.6, 22.2 and 7.4 µg/mL) of Ca-AgNPs and PEGMA capped NPs were added to each vial. Artificial sea water was added to each vial to bring the final volume to 5ml. DMSO and doxorubicin (200 µg/mL) were used as a negative and positive controls, respectively. Each study was conducted in triplicates. The vials were kept under illumination at room temperature. After 24 h the no of viable shrimps were counted using a 3X magnifying glass. Finney (1971) software was used to determine the lethal dose that killed 50% shrimps (LD50) (Mahmood et al., 2019).

### Alpha amylase inhibitory assay (antidiabetic activity)

Alpha-amylase inhibitory activity of Ca- AgNPs and PEGMA capped AgNPs was determined using a modification of the method of Sudha and Anjana (Sudha et al., 2011; Anjana et al., 2011). Using a test tube, the reaction mixture containing 350 µl phosphate buffer (50 mM, pH = 6.8), 70 µl alpha-amylase (10 U/ml) [SRL] and 140 µl of varying concentrations (7.4, 22.2, 66.6,200 µg/ml) of Ca-AgNPs and PEGMA capped NPs were pre-incubated at 37 °C for 10 min. Then 140 µl of soluble starch (0.05%) [HiMedia] as substrate was added and the reaction allowed to proceed at 37 °C for 15 min. The reaction was quenched by the addition of 140 µl of 1N HCl and 700 µl of iodine reagent (5 mM I_2_ and 5 mM KI, stored in amber colored bottle). The absorbance of each solution was then measured at 620 nm using a UV–Visible spectrophotometer. Each study was conducted in triplicates while acarbose at concentrations of 7.4, 22.2, 66.6, 200 µg/ml served as a standard. Negative control without samples was set up in parallel. The result is expressed as percentage inhibition, which was calculated as,

Inhibition (%) = A test –A negative control / A test * 100,

where, A is absorbance. The result is also expressed as IC_50_ value (Anjana et al., 2011; Sudha et al., 2011).

### Promastigote activity(antileishmanial activity)

*In-vitro* promastigote activity on *Leishmania tropica* KWH23, (obtained from Quaid-i-Azam University, Pakistan) was determined by a modified method (Shah, Khan & Nadhman, 2014). Briefly, in 96 well plate format, 10 µl of Ca-AgNPs and PEGMA capped NPs (from the stock solution, prepared in water), were mixed with 90 µl of leishmanial culture (Log phase promastigotes at 1 ×10^6^ cells per ml, maintained in RPMI medium supplemented with 10% FBS and 0.1% streptomycin and penicillin) such that the final concentration of the Ca- AgNPs and PEGMA capped AgNPs was 200, 66.6, 22.4, 7.4 ug/ml. DMSO and Glucantime were used as negative and positive control respectively. The plates were incubated at 24 °C for 72 h. After the incubation period was over, 10 µl of each dilution was pipetted on a Neubauer chamber and leishmanial promastigotes were counted under a microscope. Each determination was conducted in triplicate. IC_50_ values were calculated using table curve software.

## Results and Discussion

### Optimization of AgNO_**3**_/Plant ratios for *Caralluma tuberculata* stabilized AgNPs

**Figure 2:**
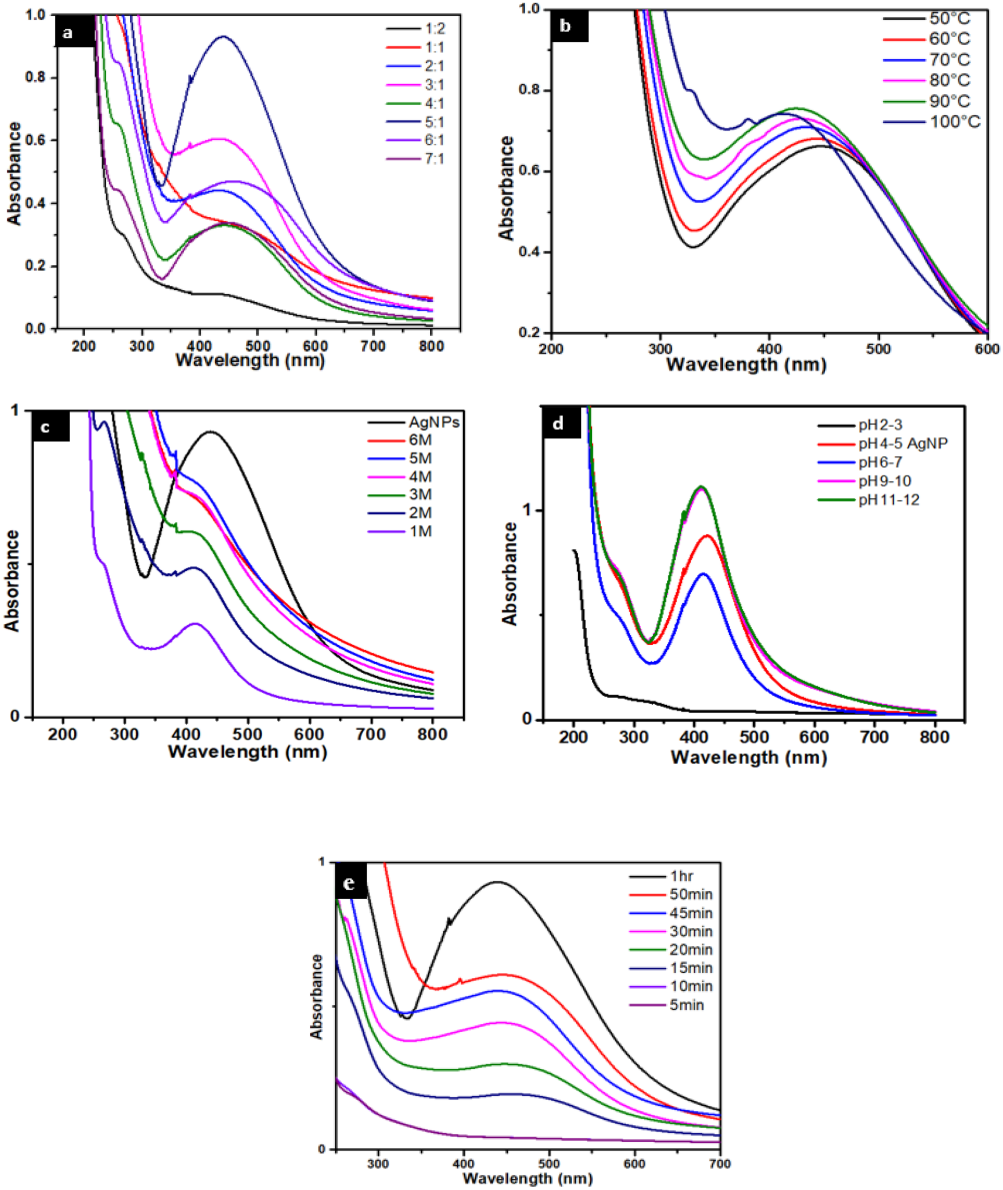
(A) UV-visible spectra of AgNPs/ *Caralluma tuberculata* having different AgNO_3_/plant ratios. (B) Effect of temperature on the stability of AgNPs. (C) Effect of salt (NaCl) on the stability of AgNPs. (D) Effect of pH on the stability of AgNPs.

The surface plasmon resonance (SPR) effect is an indication of the presence of metal nanoparticles, because the small metal nanoparticles exhibit absorption of visible electromagnetic waves through collective oscillation of conduction electrons at the surface (Link & El-Sayed, 2000). To study the presence of AgNPs UV-Vis spectroscopy was used. Figure 2A indicates the UV-Vis absorbance spectrum of AgNPs, showing a peak linked with SPR at 450 nm (Gloria et al., 2017). Optimization study was performed by carrying out different reactions by varying the amounts of salt to plant ratio. The best optimized ratio was found to be 5:1 which was then selected for further studies.

### Stability of AgNPs stabilized with *Caralluma tuberculata*

The effect of temperature was also studied and it is found that temperature affects the method of silver reduction. The solution of AgNPs were heated at 50,60,70,80,90, 100 °C for 1.5 h in a silicon oil bath. Less pronounced surface plasmon resonance peaks were observed at lower temperature and a light reddish brown color was formed. Notably, when the temperature was increased (60, 70, 80, 90, and 100 °C) more intense surface plasmon resonance peaks were observed and the color of the reaction mixtures also became dark brown (Fig. 2B). At about 90 °C maximum SPR peak was observed. It is supported in advance that on rising temperature, the reactants are consumed unexpectedly which results in the synthesis of nanoparticles (Park et al., 2007). Also blue shift was observed at higher temperature showing the formation of small size nanoparticles. At 100 °C AgNPs aggregates because of the denaturation and breaking of bonds in proteins, capping at high temperature. Due to aggregation of NPs absorbance decreases and broad peak width was observed. Thus 90 °C was found to be an optimum temperature for maximum yield of AgNPs (Amin et al., 2012).

The stability of AgNPs capped with *Caralluma tuberculata* was determined at different salt concentration ranging from 1 up to 6M. The addition of salt solutions to a colloidal solution of AgNPs has the effect of shielding the surface charge of these AgNPs and leads to consequent decrease in inter-particle distance (Kumar, 2013) with particle aggregation (Fig. 2C)*.* In fact this means that stability of AgNPs is adversely affected by the concentration of the salt used. te greater the salt concentration the greater the degree of aggregation of AgNPs (Naz et al., 2013; Bastús et al., 2014).

The overall stability of prepared AgNPs can be pH dependent. These NPs were surprisingly stable in basic medium, moderately stable in neutral medium while unstable in acidic medium. The effect of pH (5:1) on the size and shape of AgNPs was studied and it was observed that NPs aggregation seems to surpass the nucleation method in acidic conditions. At high pH, even though notable numbers of nuclei formed, as opposed to aggregation this led to the synthesis of more of NPs with smaller diameter (Fig. 2D). Repulsive forces dominate and as a result particle aggregation is reduced due to high concentration of hydroxyl ions on the surface of NPs at high pH. The optimum pH is between 11–12 (Iravani & Zolfaghari, 2013).

The formation of as a function of time were also studied. It was found that the reaction was completed within one hour (Fig. 2E). This was done by fluctuating the time taken for the formation of silver nanoparticles. The intensity of the peak is a function of contact time therefore it increases with increase in time. Contact time is one of the parameters that controls the size of silver nanoparticles. It can be inferred that at between 5 to 20 min, the SPR band is broadened because of the slow conversion of silver ion to zero valent silver nanoparticles. Increasing the contact time enhances excellent plasmon band formation because large no of Ag^+^ has been converted to Ag^0^. The maximum peak is obtained after 1 h completion of reaction (Fig. 2E) (Nasrollahzadeh et al., 2020).

### UV-Vis spectra of polymer capped with *Caralluma tuberculata* AgNPs

**Figure 3:**
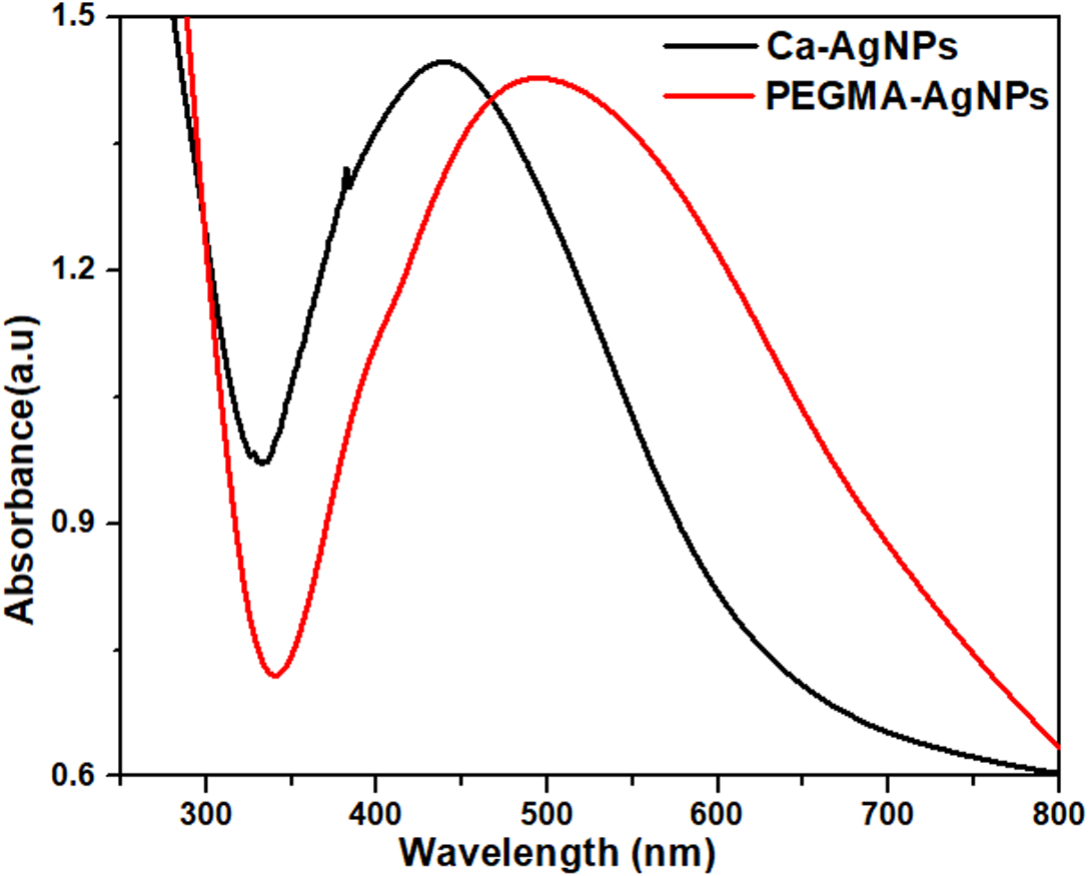
UV-visible spectra of Ca-AgNPs and PEGMA-AgNPs.

PEGMA is known to increase the stability and biocompatibility of the nanoparticles and has been used to coat the nanoparticles directly after preparation. The spectra of the Ca-AgNPs and the PEGMA capped AgNPs are compared in Fig. 3. After coating with PEGMA, the maximum emission peak of the polymer hybrid shifted to a longer wavelength (520 nm) than that of AgNPs in water (440 nm). This phenomena is consistent with previous reports (Rahme et al., 2013) due to a change in the dielectric constant at the NPs surface. The successful coating of AgNPs was confirmed by dynamic light scattering (DLS) and zeta potential (ZP) measurements.

### FTIR Spectra of *Caralluma tuberculata*, Ag Capped *Caralluma tuberculata*

**Figure 4:**
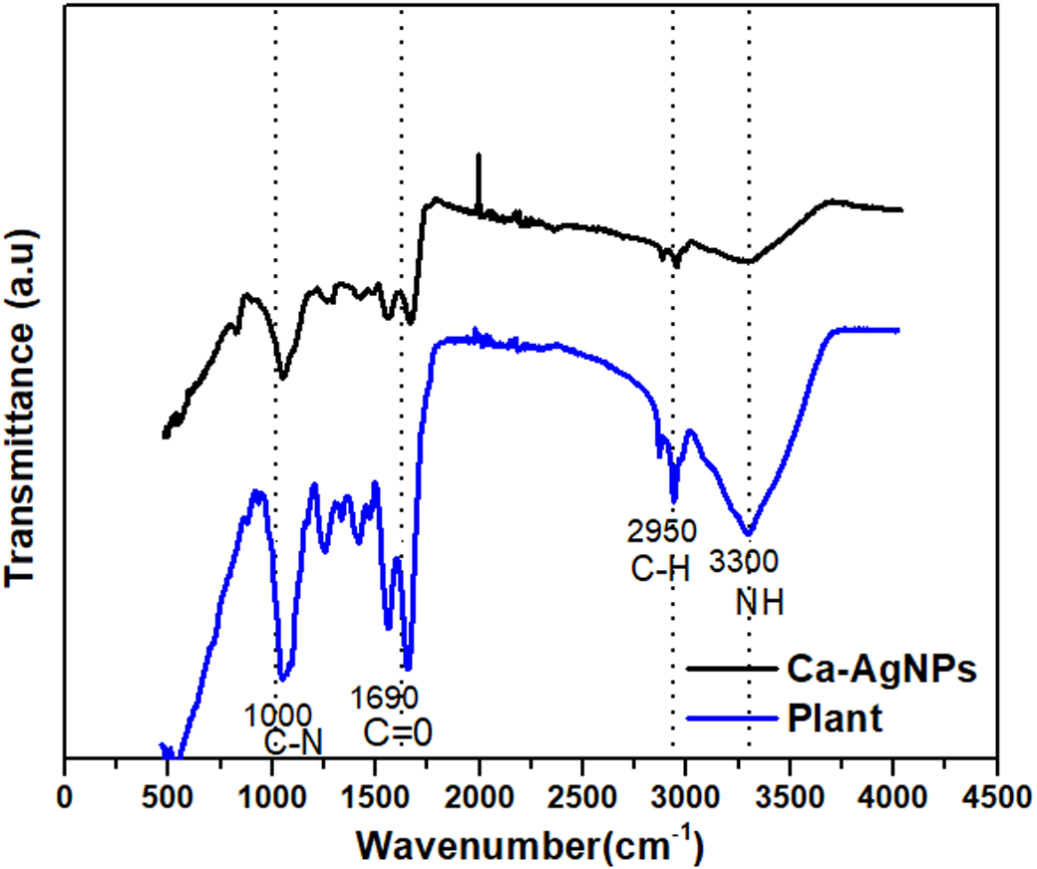
FTIR spectra of (A) *Caralluma tuberculata* plant extract and (B) Ca-AgNPs.

Typical FTIR spectra of pure *Caralluma tuberculata* and Ag-capped *Caralluma tuberculata* are represented in Fig. 4. The figure showed special IR peaks at numerous positions for the distinctive functional groups present in the samples. Furthermore a comparison of these figures clearly demonstrates successful bio fabrication of both types of nanoparticles mediated by the aid of plant extracts.

Results of FTIR spectroscopic studies for Ag-capped *Caralluma tuberculata* have show the presence of diverse chemical constituents within the silver nanoparticles (Fig. 4). Phytochemical studies on *Caralluma tuberculata* has indicated the presence of *β*- cyanin, steroids, tannins, terpenoids, amino acid and reducing sugars (Rauf et al., 2013). In the FTIR spectrum of the plant extract, there are noticeable peaks at 1600, 2950 and 3300 cm^−1^. Any shift or change in the position and intensity of these peaks in the spectrum of the plant extract can be correlated with the interaction of the functional groups of the plant extract with the silver NPs. It is possible that the functional groups especially the hydroxyl funcinal present in the extract can donate electrons resulting in the possible reduction of silver ions (Ag^+1^ to Ag^o^). FTIR spectra of the biosynthesized silver NPs showed a small shift with slight changes in some related peaks and in their intensities, suggesting that the major biomolecules from the extract were capped or bonded to the surface of silver NPs. The peak at 1690 cm^−1^ of the extract is shifted closer to a higher wave number side at 1700 cm^−1^ due to the C =O (Anandalakshmi, Venugobal & Ramasamy, 2016). The smaller peak at 2950 cm^−1^ is shifted to the higher side at 2960 cm^−1^ which corresponds to –CH stretch of alkanes. The absorption peak at 3350 cm^−1^ is related with NH (amide) stretching. The carbonyl groups showed the presence of terpenoids or flavanones which are adsorbed on the surface of metallic nano-sized particles through interaction through *π*-electrons within the carbonyl groups within the absence of sufficient concentration of chelating agents. It also shows that the carbonyl group from the protein and amino acid had stronger functionality to bind metallic nanoparticles or act as capping and stabilizing agents (Zia et al., 2016).

A vigilant examination of these figures show that the main functional groups are the same in both the representative FTIR spectra except a slight shift and the intensity is decreased. This difference in the FTIR spectra suggests the successful fabrication and capping of NPs inside the plant extract.

### FTIR spectra poly (ethylene glycol) methacrylate, Ca-AgNPs and poly(ethylene glycol) methacrylate capped AgNPs

**Figure 5:**
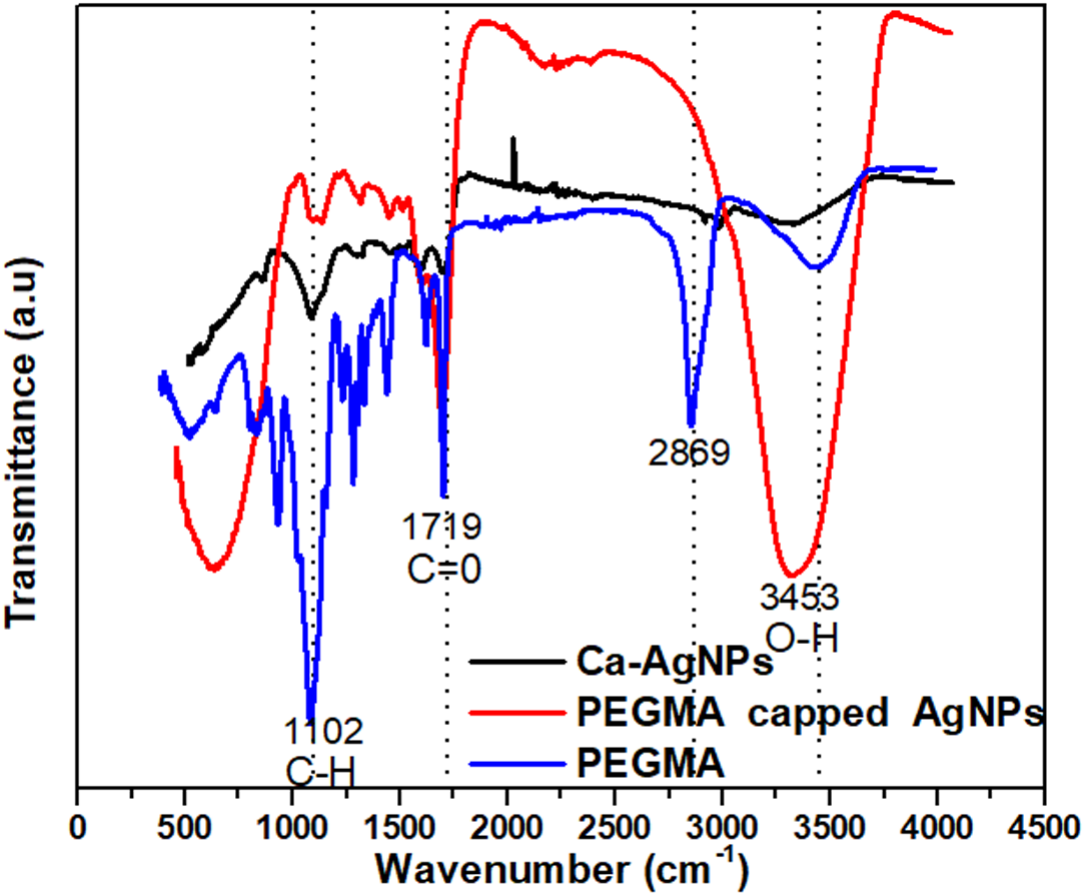
FT-IR spectra of the PEGMA, Ca-AgNPs and PEGMA-AgNPs.

The FTIR spectra of poly(ethylene glycol) methacrylate, Ca-AgNPs and AgNPs capped with poly (ethylene glycol) methacrylate (Fig. 5). The FTIR spectra of PEGMA showed adsorption peaks at 3453 cm^−1^ (O–H), 2869 and 1102 cm^−1^ (C–H), 1719 cm^−1^ (C =O) and 1628 cm^−1^ (C =C) (Fig. 5). PEGMA coating significantly altered the FTIR spectra of the AgNPs, especially the C =O and C–H peaks from the methacrylate tail and the O–H peak from PEG side chains. FTIR spectra showed that no new peak was found after the hybridization of AgNPs with PEGMA. However, upon coating a change in the position and intensity of the peak can be seen which shows that the coating process occurred *via* physicochemical adsorption without a true chemical reaction (Permadi et al., 2012).

### Scanning electron microscopy (SEM)

**Figure 6:**
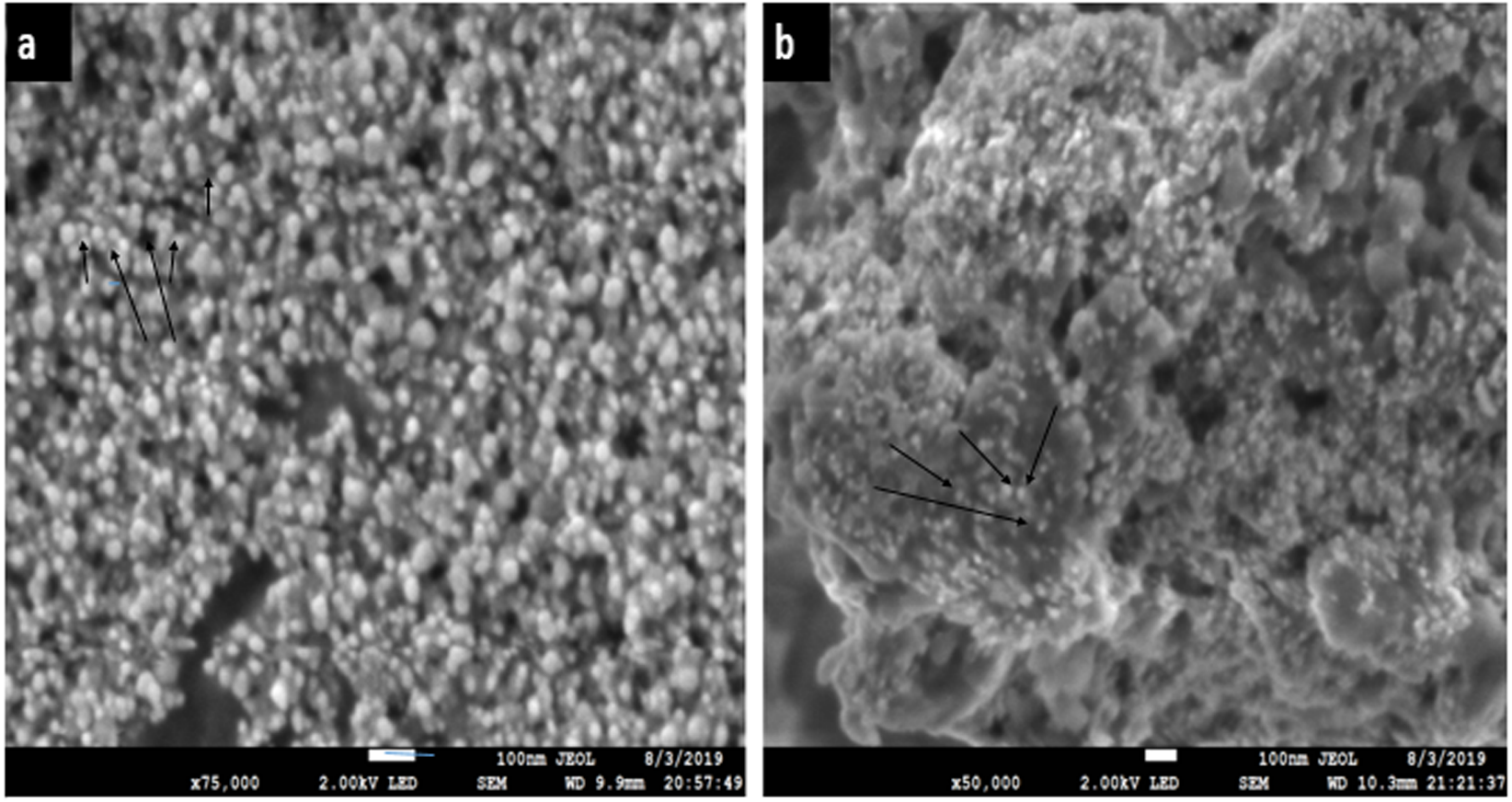
SEM images (A) of Ca-AgNPs, (B) PEGMA-AgNPs showing distribution of spherical NPs on mesh like polymer chains.

The SEM images of Ca-AgNPs (a) shows uniform particle distribution with average size of about 10 to 60 nm (Fig. 6) (Jeevanantham, Hemalatha & Satheeskumar, 2018). The SEM analysis of PEGMA coated AgNPs clearly indicates uniform distribution of the NPs in mesh like structure built by the poly(ethylene glycol) methacrylate chains as shown in Fig. 6B (Agbo, Nwofe & Ahworehe, 2017). The size of the nanocomposites is in the range of 20 to 250 nm. The SEM depicts the porous nature of the nanocomposites. The observed nature of the SEM show that the NPs-polymer composites may have interesting applications in bio-delivery and catalysis.

### Transmission Electron Microscopy (TEM) analysis of Ca-AgNPs and PEGMA capped AgNPs

**Figure 7:**
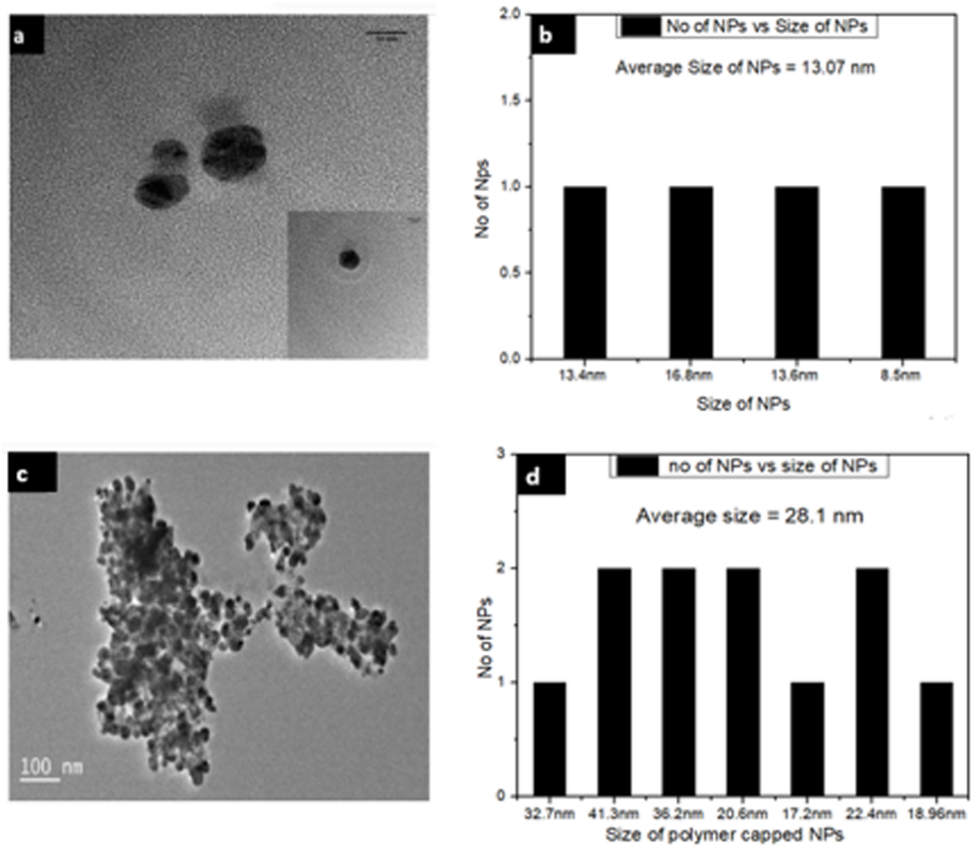
TEM images of (A) Ca-AgNPs and (C) PEGMA-AgNPs (along with histogram of (B) Ca-AgNPs and (D) PEGMA-AgNPs).

To investigate further the size and dispersion of these NPs and NPs-polymer composites, TEM analysis was also performed. The TEM images show that Ca-AgNPs formed are of different sizes but mostly spherical in shape (Fig. 7A). The average size of AgNPs was found to be 13.07 nm. The TEM images also confirm the physicochemical presence of PEGMA capping on the AgNPs. The polymer NPs image further shows the hydrophobic-hydrophilic type of attachment of the polymer with inorganic NPs (Fig. 7C). It is clear from Fig. 7C that PEGMA capped AgNPs were spherical with an average diameter of about 28.1 nm.

### Dynamic Light Scattering (DLS) and Zeta Potential (Z.P) study

**Figure 8:**
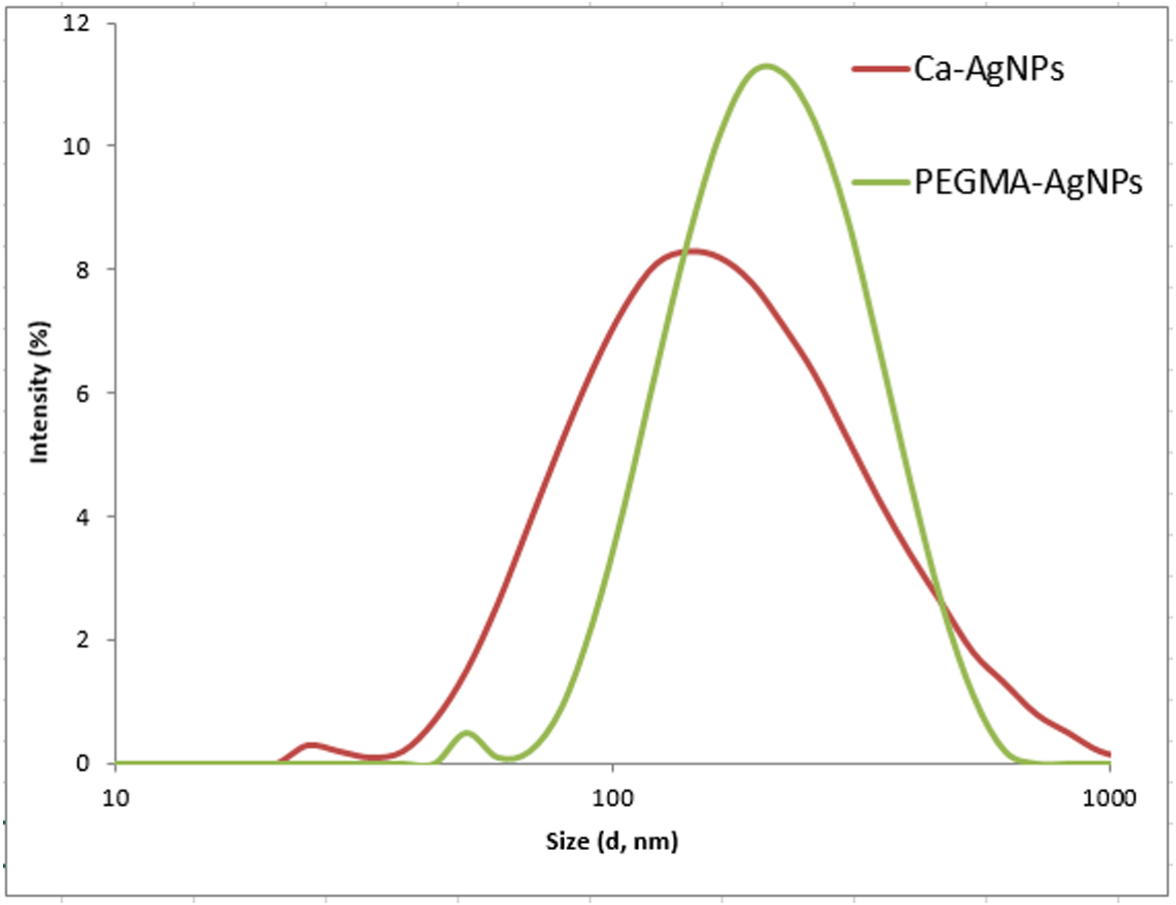
DLS of Ca-AgNPs and PEGMA-AgNPs showing increase in the size of NPs due to polymer capping.

**Figure 9:**
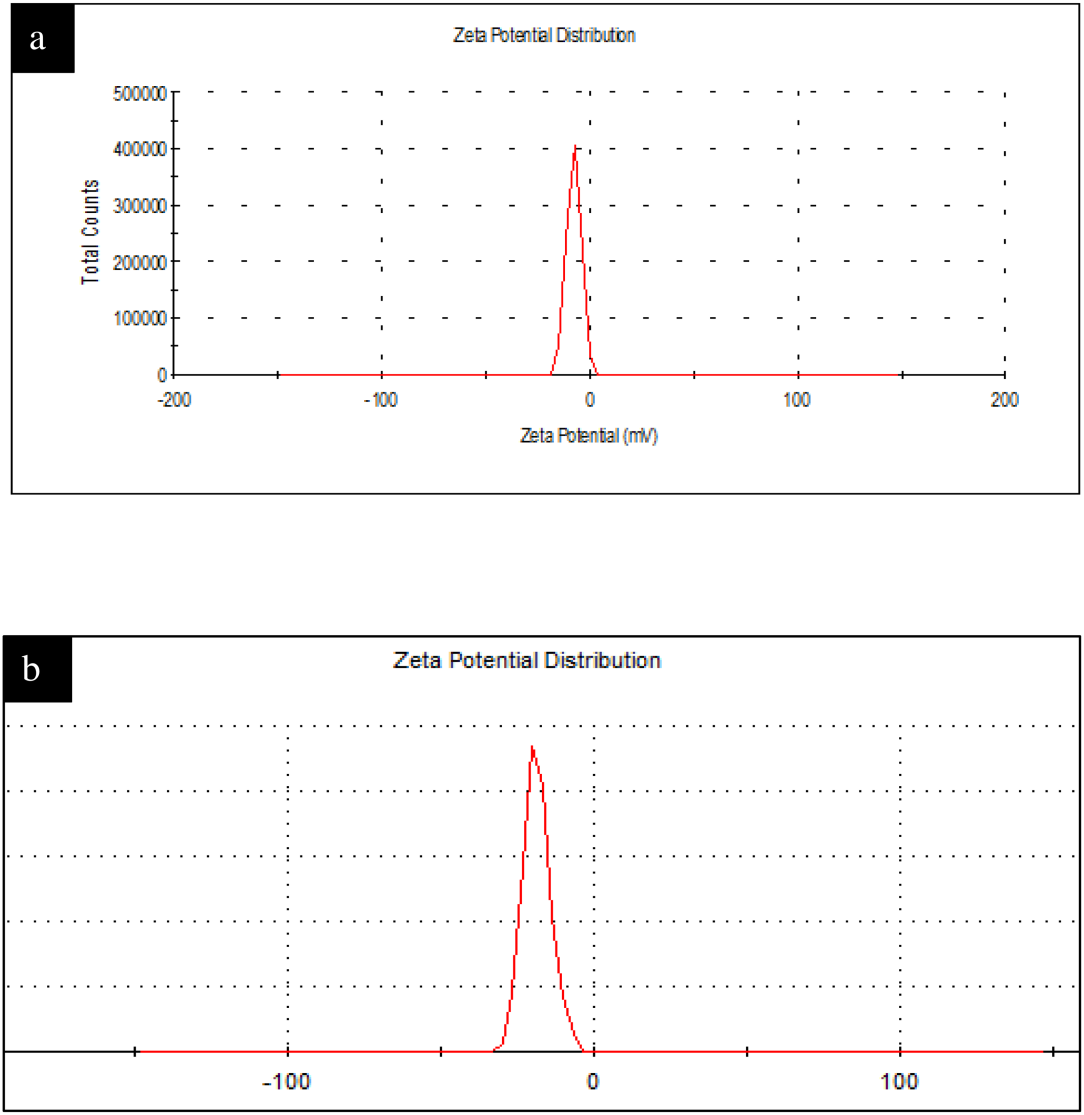
(A) Figure shows Zeta Potential Original Graph Ca-AgNPs (Zeta potential Values = −7.8) and (B) PEGMA-AgNPs (Zeta potential Values = −18.6). (A) Zeta Potential Original Graph Ca-AgNPs (Zeta potential Values = −7.8) and (B) PEGMA-AgNPs (Zeta potential Values = −18.6).

The DLS size distribution image of Ca-AgNPs ranges from 140 to 170nm and that PEGMA capped AgNPs ranges from 200 to 230 nm as shown in Fig. 8. As expected, the DLS measured size is slightly larger than the TEM size because TEM measures the exact size and doesn’t include any capping agents, but DLS measures the diameter of the particle, plus ions or molecules that are attached to the surface and moves with the AgNPs in solution (Cumberland & Lead, 2009). The particles appear larger to the instrument in comparison to TEM because of these ions or other associated molecules. Hence, the hydrodynamic diameter is always greater than the size expected by TEM (Huang et al., 2007). Nevertheless, many studies proposed the importance of hydrodynamic diameter for understanding and optimizing the size of NPs and their performance in biological assays (Huang et al., 2007; (Cumberland & Lead, 2009); Samberg, Oldenburg & Monteiro-Riviere, 2009; Singhal et al., 2011). If all the particles in suspension have a large negative or positive Z.P then they will tend to repel each other and there will be no tendency for the particles to come together (Dave et al., 2017). Negative Z. *P* value of about −18.6 mV was observed for PEGMA capped AgNPs while Ca-AgNPs exhibit Z. *P* value of about −7.8mV. The higher degree of stability of these prepared PEGMA capped AgNPs associates well with its higher surface charge as indicated by Z. *P* value (Fig. 9)(Honary & Zahir, 2013). The Z.P values also suggest that these PEGMA capped AgNPs are stable in nature and the PEGMA-AgNPs have greater stability as compared to that of Ca-AgNPs (Fig. 9).

### Evaluation of antiproliferative activity of the as-synthesized Ca-AgNPs and PEGMA capped AgNPs

**Figure 10:**
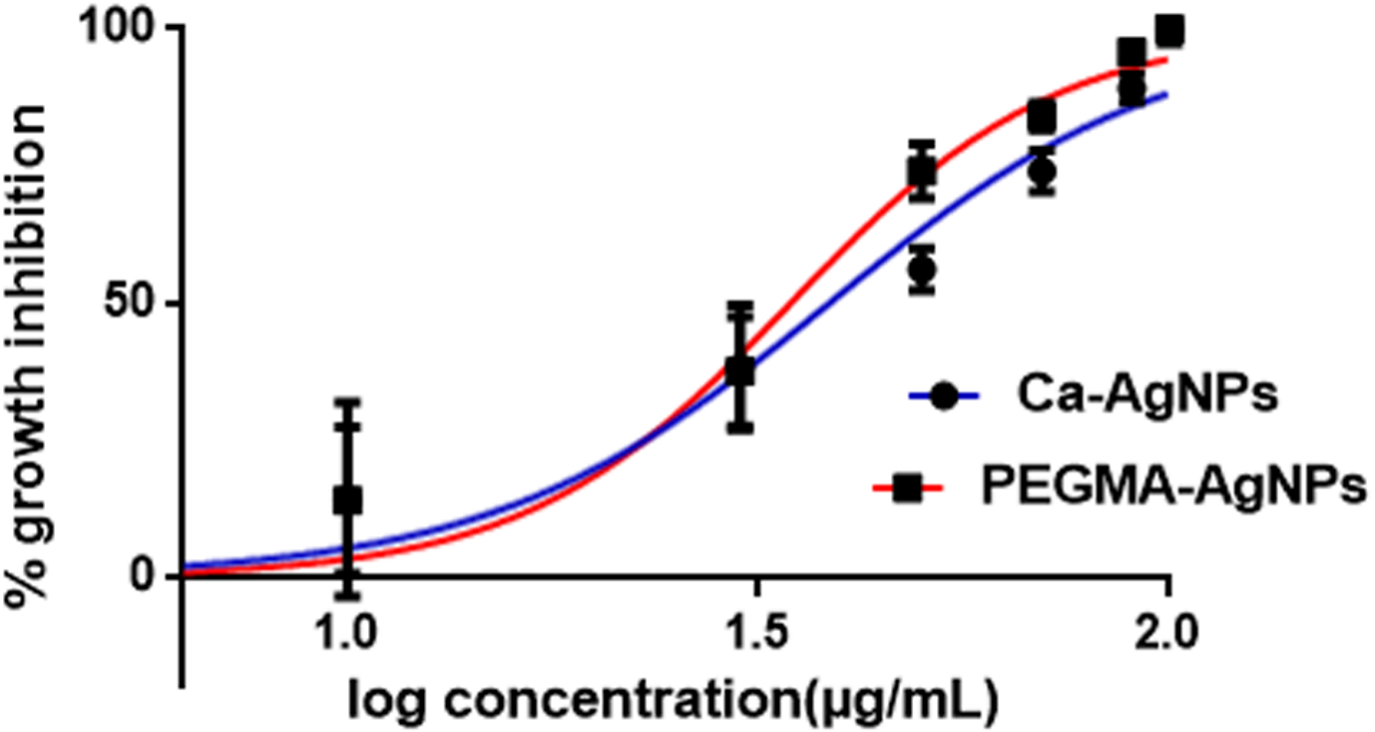
Antiproliferative activity of Ca-AgNPs and PEGMA-AgNPs.

The antiproliferative activity of Ca-AgNPs and PEGMA capped AgNPs was investigated using the 3-(4,5-dimethylthiazol-2-yl)-2,5- diphenyltetrazolium bromide (MTT) assay using PC-3 cells (human prostate adenocarcinoma cell line).To this end, PC-3 cells were treated with Ca-AgNPs and PEGMA capped AgNPs at different concentrations. After 72 h exposure to the cells, the viability of the cells was determined. Based on this result, an IC_50_ for both Ca-AgNPs and PEGMA capped AgNPs was determined. The results demonstrate a significant decrease in the number of cancer cells for PEGMA capped AgNPs compared to that of Ca-AgNPs. The IC_50_ values of Ca-AgNPs, PEGMA capped AgNPs were 38 and 34 µg/mL, respectively (Fig. 10, Table 1) which shows that the PEGMA capped NPs gave an increased IC_50_ value compared with bare NPs, but still fell well within the proper range of most anticancer drug candidates in terms of IC_50_ values. The obtained hill slopes of Ca-AgNPs and PEGMA capped AgNPs were 2.122 and 2.690, respectively (Ding et al., 2019). The obtained hill slopes also indicated that PEGMA-capped NPs exhibited positive cooperativity, whereas bare NPs exerted negative cooperativity of binding. The difference in binding behavior between polymer capped NPs and bare NPs could be ascribed to the incorporation of NPs within the polymer matrix. Furthermore, NPs was trapped within the polymer matrix and it could be slowly released to the tumor tissue, thus significantly lowering the cytotoxicity compared to Ca-AgNPs (Evangelatov et al., 2016; Akter et al., 2018). This result convincingly demonstrates that the as-prepared PEGMA capped NPs is promising antiproliferative agent.

### 2,2-diphenyl-1-picryl-hydrazyl-hydrate (DPPH) antioxidant assay

**Figure 11:**
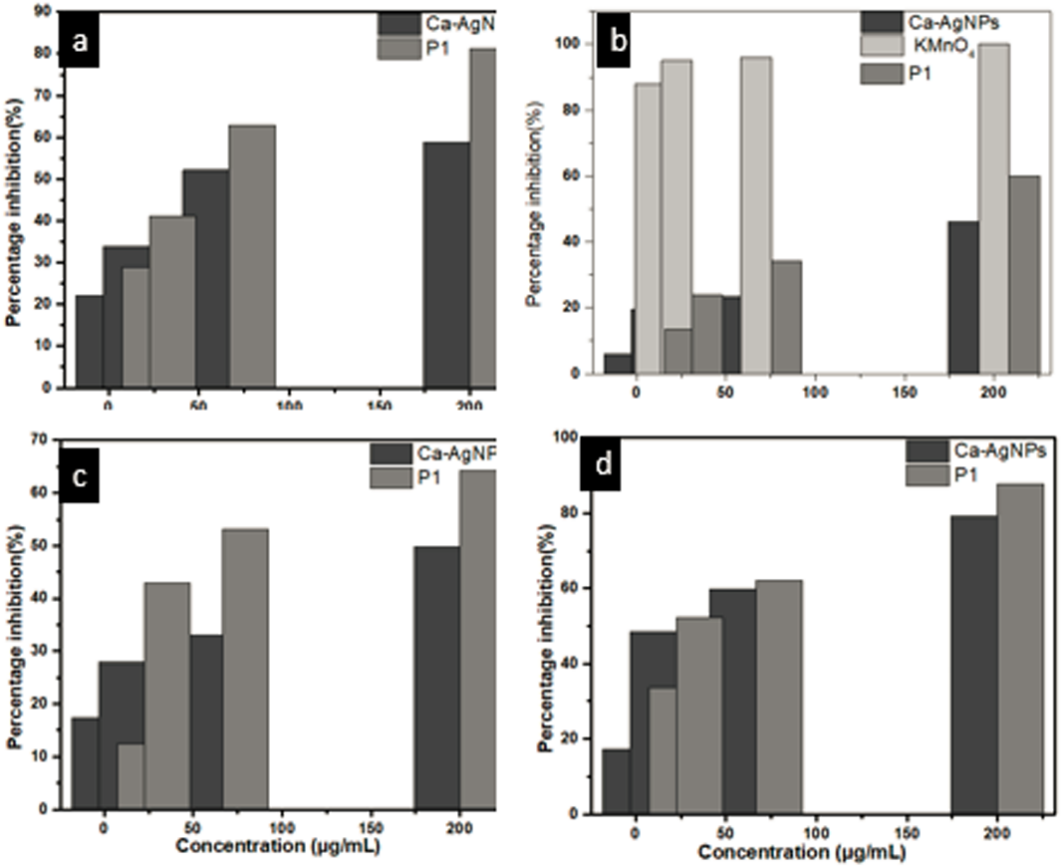
(A) Antiradical, (B) brine shrimp, (C) *α*-amylase and (D) antileishmanial assay of Ca-AgNPs and PEGMA-AgNPs at different concentrations.

DPPH radical scavenging assay was investigated for the evaluation of antioxidant potential of Ca-AgNPs and PEGMA capped AgNPs. The percentage inhibition was increased in a dose dependent manner for both Ca-AgNPs and PEGMA capped AgNPs. For lowest concentration (7.4 µg/mL) of Ca-AgNPs the percentage inhibition was 22.12 which increases to 58.72 when the concentration was increased to 200 µg/mL.However, for PEGMA capped AgNPs the percent inhibition values recorded were 28.93 for the concentration of 7.4 µg/mL and 81.18 for the concentration of 200 µg/mL, these values specify the better antiradical potential of synthesized PEGMA capped AgNPs than the Ca-AgNPs as shown in Fig. 11A. Percentage inhibition of Ca-AgNPs / PEGMA capped AgNPs was calculated by formula at different concentrations *i.e.,* 200, 66.6, 33.3 and 7.4 µg/mL and then IC_50_ value (the concentration of Ca-AgNPs / PEGMA capped AgNPs at which 50% inhibition is achieved) was calculated by graphical method by using table curve software. The synthesized PEGMA capped AgNPs displayed better antioxidant activity in comparison to Ca-AgNPs. The antioxidant capability of PEGMA has recently been reported (Jiang et al., 2015). It is postulated that the antioxidant capability of PEGMA is higher due to presence of carbonyl group (Javed et al., 2017). NPs itself bears free radical scavenging activities due to presence of phenolic compounds (Markus et al., 2017), while capping of PEGMA enhances the capabilities due functional group present on the surface.

**Table 1:** IC_50_ and R squared values of Ca-AgNPs and PEGMA capped AgNPs.

S.No.	**IC**_**50**_	**R squared**
**Ca-AgNPs**	**38**	**0.9113**
**PEGMA capped AgNPs**	**34**	**0.9568**

### Brine shrimp cytotoxic assay

The cytotoxic potential of Ca-AgNPs and PEGMA capped AgNPs were evaluated by the brine shrimp bio-assay. The IC_50_ values on the cytotoxicity of the prepared PEGMA capped AgNPs was 138.8 µg mL^−1^ (Table 2) whereas the highest mortality was witnessed at 200 µg mL^−1^ concentration. Lethality was directly related to the concentration of Ca-AgNPs as well as that of PEGMA capped AgNPs (Fig. 11B). The IC_50_ value also suggests that PEGMA capped AgNPs have better cytotoxic potential than Ca-AgNPs. This may be due to PEGMA capping on the NPs. It was reported that PEGMA-500 showed significant cytotoxicity, so capping of the polymer on the NPs enhances its cytotoxicity (Liu et al., 2017). The mechanism behind cytotoxic activity against different human cancer cell lines is considered as blockage of cellular oxidative defenses and apoptosis through production of reactive oxygen species (Vinardell & Mitjans, 2015).

### Alpha-amylase inhibitory assay

A dose-dependent increase was observed in percentage inhibitory activity against *α*-amylase enzyme. At a concentration of 7.4 µg/mL Ca-AgNPs showed a percentage inhibition 17.29 and for 200 µg/mL, it was 79.20 (Fig. 11C). Also this inhibition by PEGMA capped AgNPs (at concentration ranging from 7.4 µg/ml to 200 µg/mL) was found to be 33.65 and 87.65, respectively. The IC_50_ values of both Ca-AgNPs and PEGMA capped AgNPs are shown in (Table 2). It is clear that PEGMA capped AgNPs demonstrated high inhibitory activity against *α*-amylase as compared to the Ca-AgNPs [30]. The reason behind the improvement of antidiabetic activity in PEGMA capped AgNPs was due to the increased stability of the NPs due to polymer capping and significant movement of atoms towards the outer surface of poly(ethylene glycol) methacrylate as a result of which there is increased interaction between the polymer and the drug. Also polymer capped AgNPs interact easily with the drug as compared to bare NPs (Ali et al., 2016b; Ali et al., 2016a).

**Table 2:** Percentage DPPH inhibition, percentage mortality of brine *shrimp Artemia nauplii*, *α*- amylase and promastigote assay of Ca-AgNPs and PEGMA capped AgNPs.

**Percentage DPPH inhibition**		**Percentage mortality of brine *shrimp Artemia nauplii* **			***α*-amylase**		**Viability (%) of** **promistegote**	
**Concentration (µg/mL)**	**Ca-AgNPs**	**PEGMA- AgNPs**	**Ca-AgNPs**	**PEGMA- AgNPs**	**Ca-AgNPs**	**PEGMA- AgNPs**		**Ca-AgNPs**	**PEGMA- AgNPs**
7.4	27.73	27.92	5.87	13.49	17.29	33.65		14.45	16.42
22.4	31.11	39.87	19.45	23.83	48.43	52.22		19.34	32.1
66.6	52.93	53.11	23.38	34.19	59.82	62.14		33.02	46.2
200	62	70.23	46.09	59.91	79.2	87.65		47.24	68.23

### Promastigote activity (Antileishmanial activity)

In contrast to controls, the growth rate of promastigotes was inhibited at different concentrations of Ca-AgNPs and PEGMA capped AgNPs in a dose-dependent manner. The calculated IC_50_ values of Ca-AgNPs was 208.49 µg/mL (Fig. 11D). Similarly, the IC_50_ values of PEGMA capped AgNPs was 40.4 µg/mL, which are significantly (*P* < 0.05) higher than the measured IC_50_ values for Ca-AgNPs against *L. tropica*, reflecting more active leishmanicidal effects of PEGMA capped AgNPs as compared with bare Ca-AgNPs upon promastigotes of *L. tropica* (Allahverdiyev et al., 2011) Table 2.

### Catalytic activity of Ca-AgNPs and PEGMA capped AgNPs for the removal of toxic compounds

**Figure 12:**
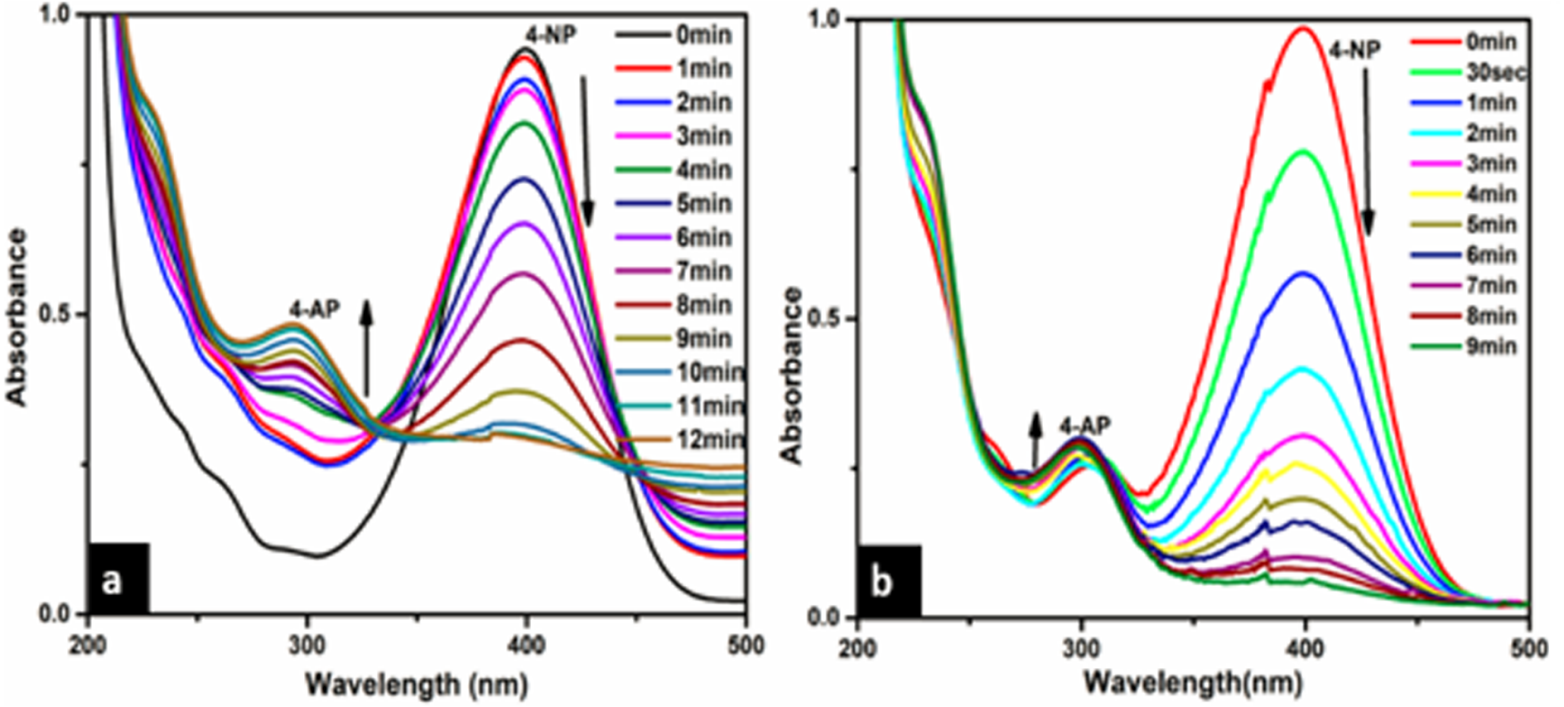
Time dependent UV-Vis absorption spectra for the catalytic reduction of 4-NP by NaBH_4_ in the presence of (A) Ca-AgNPs and (B) PEGMA-AgNPs respectively.

The catalytic reduction of 4-NP to 4-AP in the presence of NaBH_4_ as a reducing agent was taken as a model reaction to evaluate the catalytic activity of (a) Ca-AgNPs and (b) PEGMA capped AgNPs. The progress of the reaction was monitored by UV-visible spectroscopy *via* its absorption at an interval of about 1 min (Fig. 12). Initially, 4-NP shows its original absorption peak at 318 nm, after the addition of NaBH_4_ solution the formation of 4-aminophenolate ions takes place and the absorption peak shifts to 400 nm. After the addition of (a) Ca-AgNPs and (b) PEGMA capped AgNPs as catalyst, the percentage of conversion of 4-NP to 4-aminophenol (4-AP) was observed with the decrease in the intensity of absorption peak at 400 nm and appearance of a new peak at 300 nm, indicating the formation of the reduction product 4-AP. No significant change in color or intensity of absorption peak was observed in control experiments (absence of NPs and PEGMA capped NPs) during a period of 24 hrs. indicating slow reduction without addition of NPs and PEGMA capped AgNPs (Liu & Yang, 2011; Sravanthi, Ayodhya & Swamy, 2019). It was also found that the synthesized PEGMA capped AgNPs exhibits high catalytic activity for the reduction of 4-NP to 4-AP 4-NP as compared with the bare NPs.

**Figure 13:**
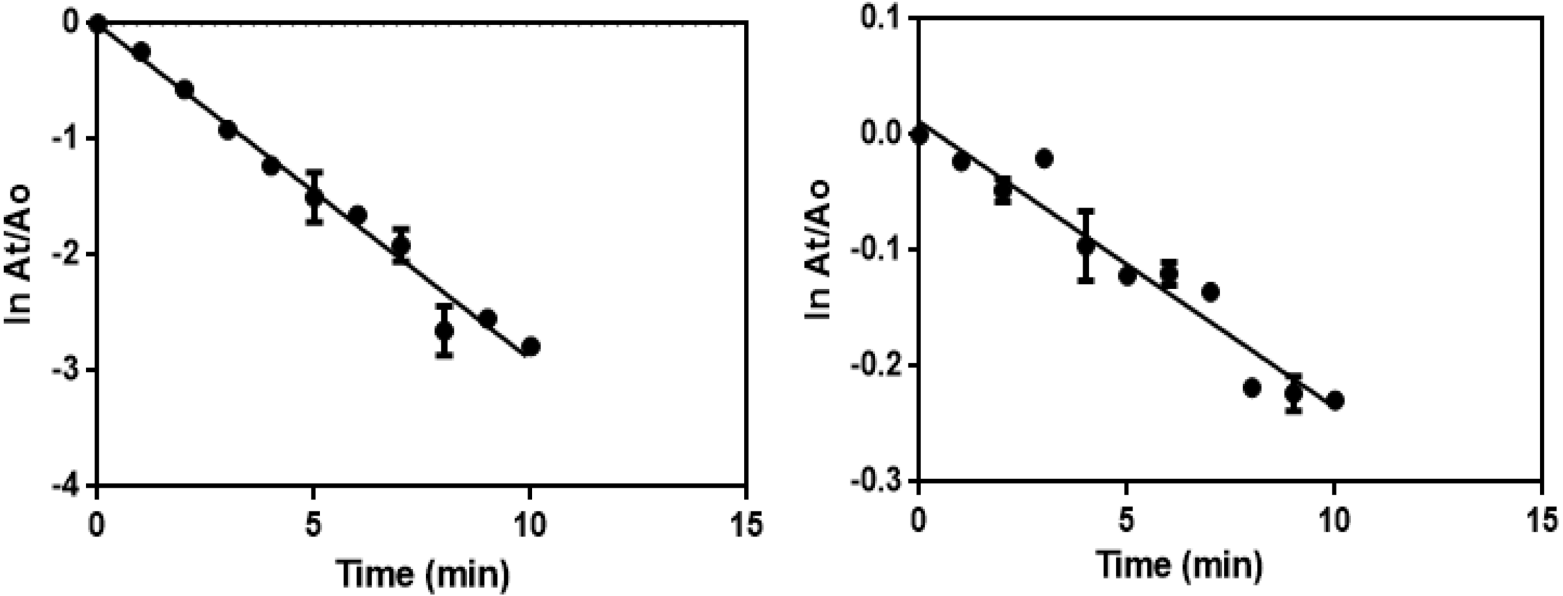
First order kinetics plot for the catalytic reduction of 4-NP by NaBH_4_ in the presence of (A) Ca-AgNPs and (B) PEGMA-AgNPs, respectively.

The excess NaBH_4_ is used to maintain alkaline condition and reduce the degradation of borohydride ions. The catalytic reduction of 4-NP using the synthesized green catalyst (Ca-AgNPs and PEGMA capped AgNPs) in the presence of excess NaBH_4_ can be fitted to a pseudo-first-order kinetic equation given by the following equation: lnCt/C0 = lnAt/A0 =-kt where *C*_0_ is the initial concentration of 4-NP, *C*_t_ is the concentration of 4-NP at a reaction time t, A_0_ is the absorbance at time t = 0 s and A_t_ is the absorbance at time t. The linear plot of lnAt/A0 against time (Fig. 13) gives the rate constant (k) which are summarized in Table 2 (Sravanthi, Ayodhya & Swamy, 2019).

## Conclusion

We have reported a green and simple procedure for the synthesis of PEGMA coated AgNPs at atmospheric pressure and room temperature. The whole method is said to be “green” because of the reduced energy consumption (reaction at atmospheric pressure and room temperature). First the extract of *Caralluma tuberculata* plant was used to stabilize and reduce the silver salt. Then *Triton* X-100 and PEGMA (PEGMA with Mn 526) was used to coat AgNPs by using reverse micellistion technique. Important features of the PEGMA coated AgNPs are its ability to catalytically reduce 4-NP and the fact that it is stable in water for 30 days. The overall results suggest that PEGMA capped AgNPs have better catalytic and dispersion properties compared to the bare metal particles. Also the polymer hybrid exhibits remarkable antiproliferative, antioxidant, cytotoxic, antidiabetic and antileishmanial activities.

##  Supplemental Information

10.7717/peerj.12540/supp-1 Supplemental Information 1 Concentration effect

10.7717/peerj.12540/supp-2 Supplemental Information 2 Effect of heat on NPs synthesis

10.7717/peerj.12540/supp-3 Supplemental Information 3 Effect of salt on NP synthesis

10.7717/peerj.12540/supp-4 Supplemental Information 4 Effect of pH on NP synthesis

10.7717/peerj.12540/supp-5 Supplemental Information 5 Uv of polymer and NPs

10.7717/peerj.12540/supp-6 Supplemental Information 6 FTIR analysis for plant and AgNO_3_

10.7717/peerj.12540/supp-7 Supplemental Information 7 Hybridization of Carulluma

10.7717/peerj.12540/supp-8 Supplemental Information 8 Histogram of AgNPs

10.7717/peerj.12540/supp-9 Supplemental Information 9 Histogram of polymer capped NPs

10.7717/peerj.12540/supp-10 Supplemental Information 10 Anti cancerous activities

10.7717/peerj.12540/supp-11 Supplemental Information 11 DPPH activity

10.7717/peerj.12540/supp-12 Supplemental Information 12 Cytotoxic activities

10.7717/peerj.12540/supp-13 Supplemental Information 13 Anti diabetic activity

10.7717/peerj.12540/supp-14 Supplemental Information 14 Antilaschminial activities

10.7717/peerj.12540/supp-15 Supplemental Information 15 Silver NPs catalysis

10.7717/peerj.12540/supp-16 Supplemental Information 16 Catalysis of polymer capped NPs

10.7717/peerj.12540/supp-17 Supplemental Information 17 Straight line of NPs

10.7717/peerj.12540/supp-18 Supplemental Information 18 Straight line graph of polymer capped NPs

10.7717/peerj.12540/supp-19 Supplemental Information 19 Time effect

## Additional Information and Declarations

### Data Availability

The following information was supplied regarding data availability: The raw data is available in the Supplemental Files.
